# Glucose-lowering therapy in type 2 diabetes: New hope after the EMPA-REG outcome trial

**DOI:** 10.1007/s00059-016-4427-3

**Published:** 2016-04-12

**Authors:** G. Schernthaner, G.-H. Schernthaner

**Affiliations:** Medical University Vienna, Wien, Österreich; Department of Medicine II, Division of Angiology, Medical University Vienna, Wien, Österreich

**Keywords:** Cardiovascular disease, EMPA-REG outcome, PROactive, Glucose-lowering therapy, Type 2 diabetes, Kardiovaskuläre Erkrankungen, EMPA-REG-Outcome-Studie, PROactive, Antidiabetische Therapie, Typ-2-Diabetes

## Abstract

Prevention of cardiovascular morbidity and mortality remains the key factor in the treatment of type 2 diabetes (T2DM). In the early phase of T2DM, multifactorial intervention is mandatory and glucose levels should be near normal, in particular in younger patients presenting with the highest cardiovascular risk. Anti-diabetic drugs without any risk for hypoglycaemia should be preferred in order to reduce clinical inertia and increase the long-term adherence to the treatment. In patients already presenting with cardiovascular disease, the best outcome may be expected with the triple oral therapy of metformin, pioglitazone, and empagliflozin, although a controlled prospective study versus insulin therapy is needed to confirm the expectation.

## Zusammenfassung

Die Verhinderung kardiovaskulärer Ereignisse und des kardiovaskulären Todes bleiben die Schlüsselfaktoren bei der Behandlung des Typ-2-Diabetes mellitus. In der Frühphase des Diabetes ist eine multifaktorielle Intervention notwendig und der Blutzuckerspiegel sollte annähernd normal sein, besonders bei jungen Patienten mit hohem kardiovaskulären Risiko. Antidiabetika ohne Hypoglykämierisiko sollten bevorzugt warden, um klinische Untätigkeit zu reduzieren und die Langzeitadhärenz der Therapie zu erhöhen. Bei Patienten mit bereits vorhandener kardiovaskulärer Erkrankung wird die beste Wirkung mit einer oralen Tripeltherapie aus Metformin, Pioglitazone und Empagliflozin erwartet, wiewohl eine kontrollierte prospektive Studie mit diesen drei Substanzen im Vergleich zu einer Insulintherapie benötigt wird, um diese Erwartung zu bestätigen.

## Introduction

The major cause of death and complications in patients with type 2 diabetes (T2DM) is cardiovascular disease (CVD). More than 60 % of all patients with T2DM die of CVD, and an even greater percentage have serious complications [1].

The impact of glucose lowering on cardiovascular complications is a worldwide debated issue. Three major studies (ACCORD, ADVANCE, and VADT) evaluated the impact of attaining euglycemia (ACCORD) or near-euglycemia (ADVANCE, VADT) in older patients with diabetes and high cardiovascular (CV) risk [2–4]. None of these studies, either individually or on pooled analysis, demonstrated any reduction in all-cause or CV mortality, although the meta-analyses revealed 15–17 % reductions in the incidence of non-fatal myocardial infarction in those exposed to tight glucose control [5]. A higher mortality was observed in the intensive glucose control arm of ACCORD, resulting in the premature termination of the glucose-lowering component of this study [2]. Also, the occurrence of hypoglycaemic episodes (total and major) was significantly higher in the intensive glucose control arms of all three studies [1]. In addition to hyperglycaemia, patients with T2DM often present with additional risk factors that predispose them to CVD. These include insulin resistance, obesity, hypertension, dyslipidaemia, chronic inflammation, platelet abnormalities, and chronic inflammation [6].

## Recent dramatic decline of all-cause mortality and CV death in T2DM

During the last 25 years (1976–2001) an impressive decline in all-cause (−48 %) and cardiovascular disease (CVD) mortality (−62 %) rates among both men and women with diabetes mellitus was observed in the Framingham study versus the period of 1950–1975 [7]. The implementation of the multifactorial CV risk factor management (blood pressure and lipid lowering) resulted in an enormous improvement in the prognosis of T2DM treated in developed nations (US, EU countries, Canada, Australia). Data from the Danish National Diabetes Register showed that the mortality rate of T2DM patients decreased by 40 % from 1997 to 2007 [8]. Similarly, the excess mortality of patients with T2DM in Canada (Ontario) and in UK (THIN database), decreased by 44 and 43 % respectively from 1996 to 2009 [9]. In addition data from Australia showed that the age-standardized mortality rates decreased from 9.4 to 5.5 per 1,000 patient years from 1997–2010 [10]. However, in many countries both men and women with T2DM continue to remain at a higher risk of all-cause and CVD mortality than those without DM despite risk-reduction strategies that include lowering of cholesterol and blood pressure, and smoking cessation [11].

## Primary prevention of CVD: relevance of multifactorial intervention including diabetes control

**Fig. 1:**
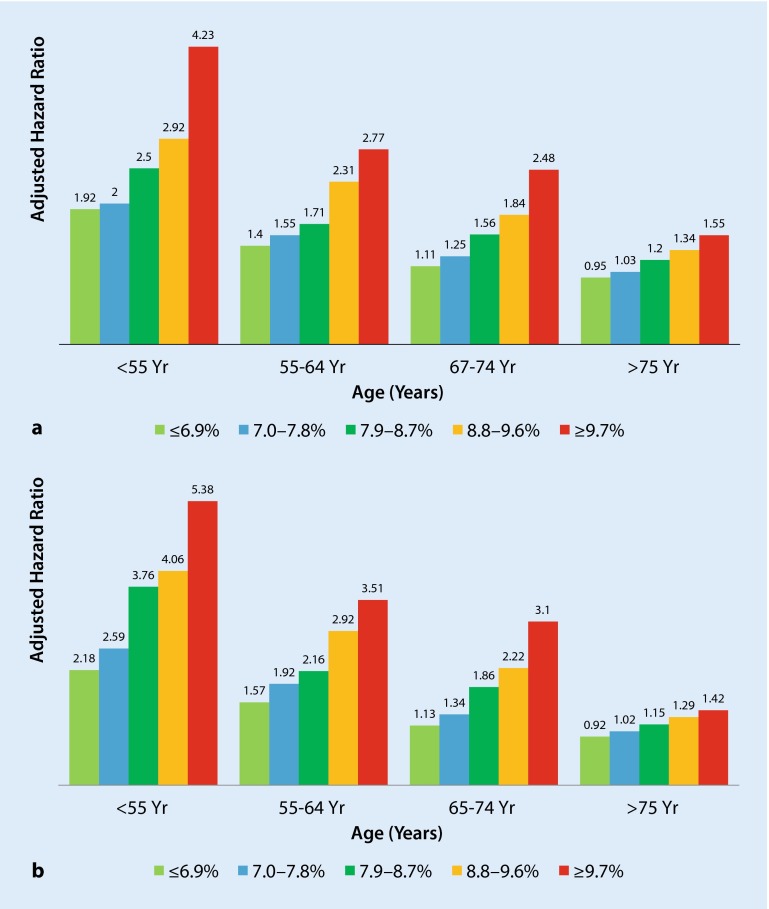
**a** Death from any cause among patients with T2DM versus controls in relation to age and mean glycated haemoglobin levels. **b** CV death among patients with T2DM versus controls in relation to age and mean glycated haemoglobin levels. *p*-values for the interaction term between time-updated mean glycated haemoglobin or renal disease status and time-updated age categories were less than 0.001 in all models

Unfortunately, we do not have any long-term multifactorial intervention study in newly diagnosed T2DM patients. A recently published nationwide study [12], which included 435,369 patients with T2DM from the Swedish National Diabetes Register and for each patient five controls randomly selected from the general population and matched according to age, sex, and county (total number of controls *n* = 2,117,483), showed that the excess mortality in T2DM was substantially higher with worsening glycaemic control, severe renal complications, impaired renal function, and younger age. The included patients had the following characteristics: mean age 65.8 years, age at diagnosis of T2DM 60.2 years, 44.9 % women, mean BMI: 29.8 kg/m^2^ and duration of diabetes 5.6 years. After a follow-up of 5 years 77,117 of 435,369 patients with T2DM (17.7 %) died, as compared with 306,097 of 2,117,483 controls (14.5 %) (adjusted HR, 1.15; 95 % CI 1.14–1.16) [12]. The overall excess risk of death from any cause was very low as compared with earlier reports, when the analysis was adjusted for age and sex and the excess risk decreased to 15 % when the analysis was further adjusted for coexisting diseases. The relatively low mortality in the Swedish T2DM patients is probably due to aggressive treatment with statins and blood-pressure medications and relatively good diabetes control. Mean blood pressure was 140/78 mmHg, mean HbA1c was 7.1 %, and mean LDL was 2.94 mmol/l. The excess risk of death ranged from 30–40 % among patients 65–74 years of age, as compared with controls in the same age group, whereas the excess mortality was 100–200 % among those younger than 55 years of age, as compared with controls. Remarkably, all-cause mortality (Fig. 1a) and CV death (Fig. 1b) were closely related to glycaemic control (HbA1c) in all age groups. However, the relationship was much stronger in younger patients and less pronounced in elderly patients. Remarkably, patients 65–74 years of age with normoalbuminuria and an HbA1c of ≤ 6.9 % had a lower risk than the controls. Similarly, the risk was also lower among patients ≥ 75 years of age with an HbA1c of ≤ 7.8 % than among the controls, but the risk was substantially higher among patients younger than 55 years of age than among the controls, despite an HbA1c level in the target range and normoalbuminuria.

## Multifactorial risk factor control is less performed in patients without CVD

A recent large study [13] including about 860,000 patients assessed the incidence of major CV hospitalization events and all-cause deaths among adults with diabetes with or without CVD associated with inadequately controlled diabetes (HbA1c), high LDL-cholesterol (LDL-C), high blood pressure (BP), and current smoking. Inadequate risk factor control was classified as LDL-C ≥ 100 mg/dl, HbA1c > 7 %, BP ≥ 140/90 mmHg, or smoking. Interestingly, compared with those without baseline CVD, those with baseline CVD had better control of smoking (8.0 vs. 9.8 %), HbA1c ≥ 7 % (42 vs. 53 %), and LDL-C ≥ 100 mg/dl (38 vs. 58 %), and they had similar proportions of subjects with systolic/diastolic BP ≥ 140/90 mmHg (23 vs. 21 %). Mean age at baseline was 59 years; 48 % of subjects were female, 45 % were white, and 31 % had CVD. Mean follow-up was 59 months. Major CV events were based on primary hospital discharge diagnoses for myocardial infarction (MI) and acute coronary syndrome (ACS), stroke, or heart failure (HF). Event rates per 100 person-years for adults with diabetes and CVD versus those without CVD were 6.0 vs. 1.7 for MI/ACS, 5.3 vs. 1.5 for stroke, 8.4 vs. 1.2 for HF, 18.1 vs. 40 for all CV events, and 23.5 vs. 5.0 for all-cause mortality. The percentages of CV events and deaths associated with inadequate risk factor control were 11 and 3 %, respectively, for those with CVD but 34 and 7 %, respectively, for those without CVD. These data demonstrate that (a) T2DM patients without CVD are not as well treated for risk factor control and (b) that the inadequate risk factor control in patients without CVD has a very negative impact on CV events and death.

## CV outcome studies with novel anti-diabetic agents in patients with T2DM

Due to the close association of CVD with T2DM and the uncertainty about the CV safety of glucose-lowering drugs, the Food and Drug Administration issued in 2008 guidance for the demonstration of CV safety for new anti-diabetes drugs [14].

**Fig. 2:**
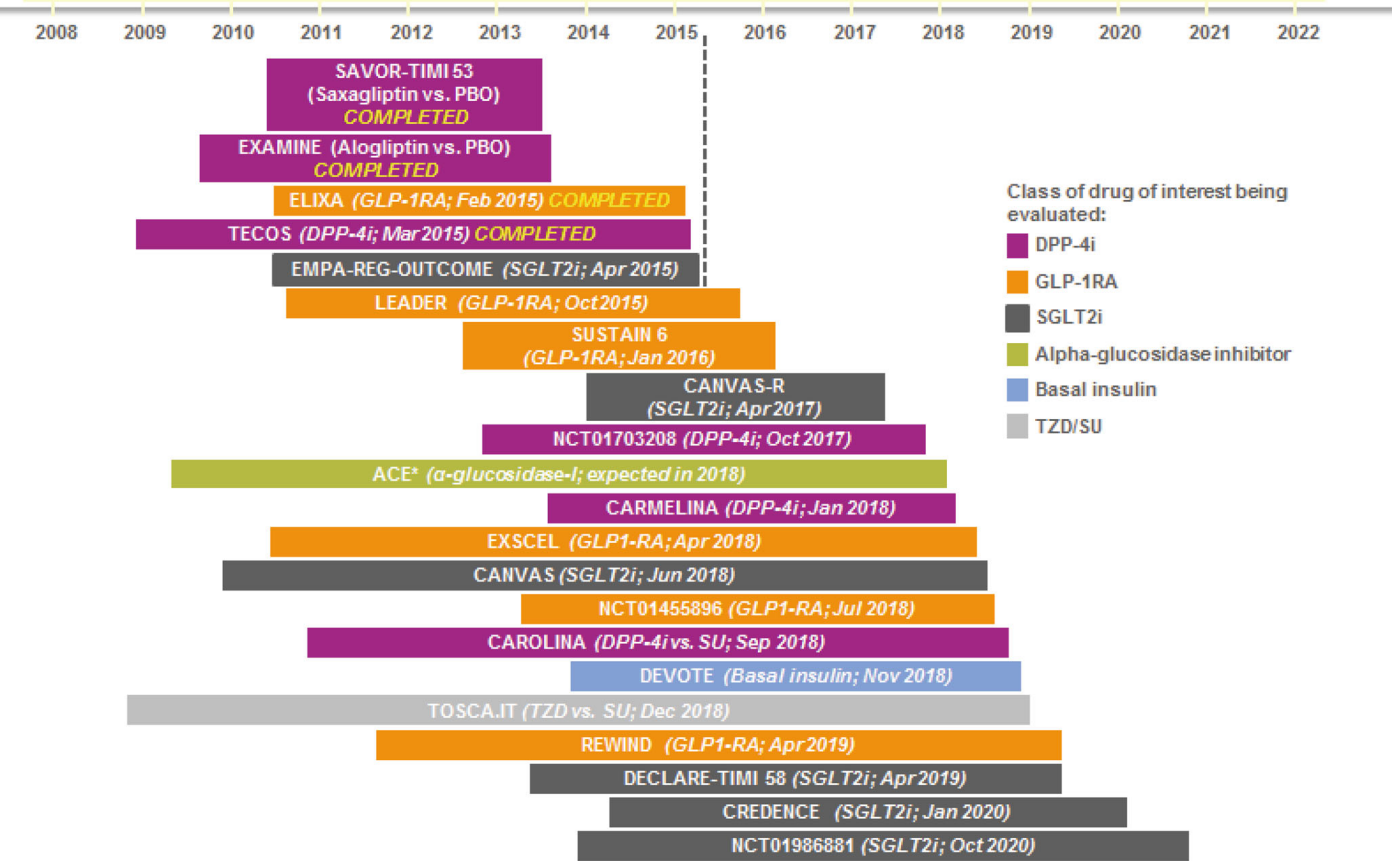
CV outcome studies in patients with T2DM: chronology of completion dates. (https://clinicaltrials.gov/ct2/home; last accessed May 29, 2015) (SAVOR-TIMI 53, NCT01107886; EXAMINE, NCT00968708; ELIXA, NCT01147250; TECOS, NCT00790205; EMPA-REG-OUTCOME, NCT01131676; LEADER, NCT01179048; SUSTAIN 6, NCT01720446; CANVAS-R, NCT01989754; ACE, NCT00829660 (*https://www.dtu.ox.ac.uk/ace/; last accessed May 29, 2015); CARMELINA, NCT01897532; EXSCEL, NCT01144338; CANVAS, NCT01032629; CAROLINA, NCT01243424; DEVOTE, NCT01959529; TOSCA.IT, NCT00700856; REWIND, NCT01394952; DECLARE-TIMI 58, NCT01730534; CREDENCE, NCT02065791; NCT01986881)

Fig. 2 summarizes all CV outcomes trials (CVOT), which will be available at the end of the year 2020). At that time all available CVOT studies will include about 180,000 patients with T2DM and CVD followed up with a novel anti-diabetic drug or placebo in addition to standard care. In the mean time we have for most of the newer glucose-lowering drugs results from CVOT, the PROactive study [15] for pioglitazone, the ORIGIN study [16] for Insulin Glargine, the SAVOR study [17] for Saxagliptin, EXAMINE for Alogliptin [18], TECOS for Sitagliptin [19] and ELIXA for Lixisenatide [20]. The design of these studies makes it almost impossible to show benefits of any novel drug. All patients were at high CV risk and had long-standing uncontrolled T2DM for 8–10 years. Treatments that might be effective for the primary prevention of CVD and have potential CV benefits in early intervention may be ineffective in the progressed stage of T2DM. In addition, most patients were receiving standard care treatments for their CVD (antiplatelet agents, including aspirin, 75–97 %; statins, 78–90 %; beta-blockers, 62–85 %; angiotensin converting enzyme inhibitor/ angiotensin receptor blocker, 79–85 %) so that any potential added CV risk reduction or secondary CVD prevention by a novel anti-diabetic drug was less likely to be observed. The published results show that most agents, with the exception of pioglitazone and empagliflozin, neither increased nor decreased major adverse CV events (CV death, non-fatal myocardial infarction, and non-fatal stroke) compared with placebo (Table 1).

**Table 1:** Effect of glucose-lowering drugs on the combined endpoint of CV mortality, non-fatal myocardial infarction and stroke

Study	Anti-diabetic Drug	HR	*p*-value
PROACTIVE	Pioglitazone	0.84 (CI 0.72–0.98)	0.02
ORIGIN	Insulin Glargine	1.02 (CI 0.94–1.11)	NS
SAVOR	Saxagliptin	1.00 (CI 0.89–1.12)	NS
EXAMINE	Alogliptin	0.96 (CI 0.80–1.15)	NS
CANVAS	Canagliflozin	1.00 (CI 0.72–1.39)	NS
ELIXA	Lixisenatide	1.02 (CI 0.89–1.17)	NS
TECOS	Sitagliptin	0.98 (CI 0.89–1.08)	NS
EMPA-REG	Empagliflozin	0.86 (CI 0.74–0.99)	0.038

*HR* hazard ratio, *CI* confidence interval, *NS* not significant

Remarkably, a significant and similar reduction of the three-point MACE (CV death, non-fatal MI, and non-fatal stroke) was found in [15] for pioglitazone (HR 0.84; 95 % CI 0.72–0.98) and for Empagliflozin (HR 0.86; 95 % CI 0.74–0.99) in the EMPA-REG outcome study [21], however with striking differences. In PROactive [15] both fatal/nonfatal stroke (HR = 0.72, *p* = 0.045) and fatal/nonfatal myocardial infarction (HR = 0.53, *p* = 0.008) were markedly reduced, whereas the small reduction of MI and the increase of stroke in EMPA-REG outcome [21] did not reach levels of significance. By contrast the impressive reduction of CV death and all-cause mortality seen in patients exposed to empagliflozin was not seen with pioglitazone.

## PROactive

The PROactive study [15] was a large prospective, randomized, double-blind, secondary prevention study that investigated the effects of pioglitazone (45 mg/day) on macrovascular outcomes in 5,238 patients with T2DM and pre-existing CVD: ~ 50 % with previous MI, 25 % with previous stroke, and 25 % with peripheral arterial disease (PAD). Treatment with pioglitazone or placebo was administered in addition to optimized standard care, which included glucose lowering, antihypertensive, lipid-altering, and antithrombotic drugs. Although the primary end point – a composite of all-cause mortality, non-fatal MI, acute coronary syndrome, stroke, major leg amputation, and coronary or leg revascularization – showed only a nonsignificant 10 % reduction in the pioglitazone arm, a significant reduction in a composite end point, comprising CV death plus non-fatal MI plus non-fatal stroke, was observed (HR 0.82 [95 % CI 0.70–0.97]) in the 3-year follow-up period [15]. Furthermore, in patients with a previous MI, pioglitazone significantly reduced the risk of subsequent MI by 28 % and acute coronary syndrome by 38 % [22]. In patients with a previous stroke, pioglitazone decreased chances of a second stroke by 48 % [23], whereas in patients with PAD no beneficial effect of pioglitazone could be noted [24]. It is well known that diabetic patients with chronic kidney disease (CKD) are at particularly high risk of CVD. In a post hoc analysis from PROactive, the effect of pioglitazone versus placebo was determined in patients with CKD [25]. Patients treated with pioglitazone were less likely to reach the composite (all-cause mortality, MI, or stroke) end point (HR 0.66 [95 % CI 0.45–0.98]) compared with placebo. In addition, two randomized head-to-head trials with glimepiride have shown that pioglitazone significantly decreased the rate of carotid intima thickness, a surrogate marker of coronary atherosclerosis [26] and slowed the progression of coronary atherosclerosis measured by IVUS [27]. The anti-atherogenic effect of pioglitazone may be mediated by the improvement of many CV risk factors [28, 29], such as increase in HDL-cholesterol, decline of triglycerides and free fatty acids (FFA), conversion of small dense LDL particles to larger, more buoyant, less atherogenic ones; improvement of endothelial dysfunction; increase of adiponectin and reduction of PAI-1, CRP, and TNFα, and reduction of insulin resistance and visceral fat.

The clinical use of pioglitazone is limited by the risk of adverse events, including weight gain, fluid retention, CHF and bone fractures [30]. In the PROactive study [15] 5.7 and 4.1 % of pioglitazone and placebo patients, respectively, were admitted to hospital [31]; however, mortality rates due to CHF were similar (0.96 vs. 0.84 %; *p* = NS). Interestingly, fewer pioglitazone patients with serious CHF had a combined end point of death, MI, or stroke compared with placebo patients (34.9 vs. 47.2 %; *p* = 0.025). Since heart failure is an ominous sign in T2DM with a five-year mortality of ~ 50 %, it is unlikely that these individuals really had CHF. It is more likely that they had fluid retention and oedema secondary to the sodium retention effect of pioglitazone in the kidney. Concern about bladder cancer with pioglitazone has been negated by the results of a 10-year prospective Kaiser-Permanente Northern-California study [32]. In that safety study involving 193,099 T2DM patients, no association was found between bladder cancer risk and use of pioglitazone, including duration of pioglitazone use, cumulative pioglitazone dose, or time since initiation of pioglitazone. A further study [33] including 1.01 million T2DM patients with over 5.9 million person-years from six populations, reported no increased risk for bladder cancer either for pioglitazone (HR = 1.01) or rosiglitazone (HR = 1.00).

## EMPA-REG outcome

**Fig. 3:**
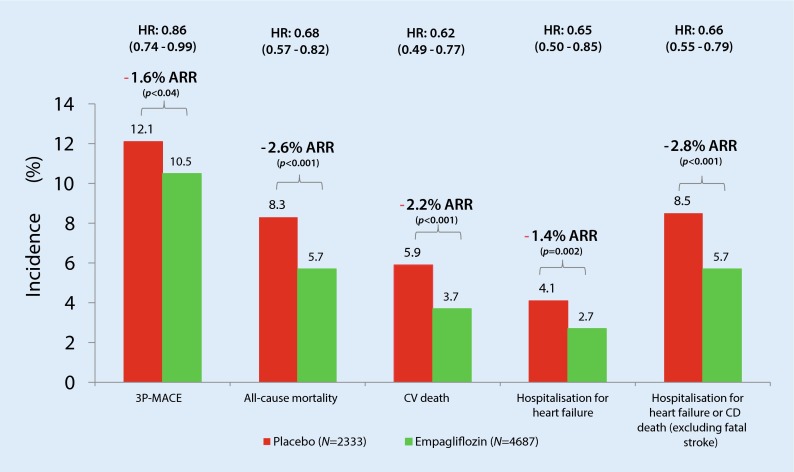
Significant Improvement of CV outcomes by Empagliflozin. *HR* hazard ratio. Indicated with 95 % confidence intervals; *ARR* Absolute risk reduction

The recently published EMPA-REG outcome trial [21] is an international, prospective, placebo-controlled clinical trial investigating the cardiovascular outcomes of empagliflozin, an inhibitor of sodium-glucose cotransporters type 2 (SGLT2), in patients with T2DM and known CVD. It is the first study to document that a glucose-lowering drug can reduce cardiovascular events in patients with T2DM. In 7020 T2DM patients with a history of CVD, empagliflozin reduced, after a median of 3.1 years, the primary MACE endpoint (CV death, non-fatal MI, non-fatal stroke) by 14 % (HR = 0.86, *p* = 0.04) and hospitalization for heart failure by 35 % (HR = 0.65, *p* = 0.002). A striking difference was observed between the three MACE endpoints: (a) for CV death, the HR (0.62) was decreased significantly by 38 %, (b) for non-fatal MI, the HR (0.87) was decreased slightly, but not significantly (*p* = 0.22) and (c) for stroke, the HR (1.24) was increased modestly, but not significantly (*p* = 0.22). Fig. 3 shows the impressive effects of empagliflozin on the absolute risk reduction of CV events in the EMPA-REG outcome study. The reduction in CV death (5.9 to 3.6 %, *p* < 0.001) was observed across all diagnostic categories (sudden death, 1.6 to 1.1 %; worsening heart failure, 0.8 to 0.2 %; acute MI, 0.5 to 0.3 %; stroke, 0.5 to 0.3 %; “other CV death”, 2.4 to 1.6 %). The latter category includes deaths that cannot be explained by any other known cause. The reduction in mortality appeared very early (< 3 months) and was observed in all subgroups, without any obvious heterogeneity. This reduction in mortality does not seem to be fully explained by the concomitant slight reductions in HbA1c, body weight, waist circumference, blood pressure, and serum uric acid levels in the empagliflozin groups versus the placebo group. The rapid reduction of mortality in empagliflozin-treated patients suggests a hemodynamic mechanism of action. The baseline BP (135.5/76.7 mmHg) was significantly reduced at 4 months (~ 5/2.5 mmHg), and temporarily correlated with the reduction in CV death and hospitalization for heart failure. A recent study [34] showed that empagliflozin reduced not only BP but had also favourable effects on markers of arterial stiffness and vascular resistance. The observation that empagliflozin has an impact on the vasculature without increasing pulse rate is interesting from a CV perspective and could be interpreted as a consequence of a relative reduction in the sympathetic nervous system tonus. It seems likely that the beneficial effects of empagliflozin to reduce CV risk and heart failure are related to the drug’s hemodynamic/cardiovascular action to reduce BP and intravascular volume, resulting in combined afterload and preload reduction.

Surprisingly, an impressive renoprotection was also observed [35] although 26 % of the patients with CVD also had CKD (eGFR < 60), usually not receiving SGLT2 inhibitors. New onset or worsening kidney disease were reduced by 39 %, new macro-albuminuria by 38 %, doubling of serum creatinine by 44 %, and incidence of end-stage renal disease by 55 %. It is important to mention that the majority of patients with CKD at baseline had stage 3a (68 %), whereas stage 3b existed in 32 %, furthermore all patients with CKD stages 4 and 5 were excluded from the EMPA-REG outcome study.

The EMPA-REG study also confirms the excellent safety profile of the SGLT2 inhibitor (SGLT2i) class of anti-diabetic agents. Empagliflozin significantly reduced HbA1c, body weight, waist circumference, and blood pressure without change in heart rate. There was no increase in the incidence of hypoglycaemia despite half of the patients were pre-treated with insulin, renal impairment, urinary tract infections, volume-related side effects, bone fractures, or thromboembolic events. Furthermore, the rate of hyperglycaemic or normoglycaemic diabetic ketoacidosis was very low and not higher in patients exposed to empagliflozin (0.035 %) versus placebo (0.020 %). Serious adverse events and adverse events leading to drug discontinuation were slightly, although not significantly lower in the empagliflozin group. As expected, the incidence of genital infections was higher in the empagliflozin group (6.4 %) vs. placebo (1.8 %).

## Individualization of anti-diabetic therapy in relation to stage of the disease and co-morbidity

The ADA-EASD consensus statement published in 2009 [36] advocated initial treatment with metformin monotherapy and lifestyle modification, followed by addition of basal insulin or a sulfonylurea if glycaemic goals are not met (tier 1 recommendations). All other glucose-lowering therapies were relegated to a secondary (tier 2) status and only recommended for selected clinical settings. The approach that all patients should have the same HbA1c target (< 7.0 %) and that all patients should follow an identical treatment algorithm was heavily criticised by an international expert group [37]. We argued for an appropriate selection of anti-diabetic drugs to individualise and optimise care with a view to sustained control of blood glucose and reduction both of diabetes complications and CV risk. In addition, we stated that diabetes guidelines might need revision to define a minimum HbA1c value, especially for patients with long-standing diabetes or established CVD. The ADA-EASD consensus statements 2012 [38] and 2015 [39] included most of our proposals.

Patients not presenting with vascular complications should have near-normoglycaemic control in association with strict CVD risk factor control as documented in the recently published Swedish population study [12]. In particular, younger patients with a poor long-term risk need HbA1c target levels < 6.5 %, which can be reached when glucose-lowering drugs are selected not inducing hypoglycaemia or weight gain. Metformin remains the optimal drug for monotherapy, its low cost, proven safety record, weight reduction or neutrality and possible benefits on cardiovascular outcomes have secured its place as the favoured initial drug choice [40–42]. In second line, DPP-4 inhibitors are now widely used, since these drugs are well tolerated by the majority of patients, even in the elderly and renal-impaired patients [43–46]. DPP-4 inhibitors improve glycaemic control with similar efficacy to sulphonylurea, but do not usually provoke hypoglycaemia or weight gain, are relatively free from adverse effects, and have recently been shown not to increase CV risk in large prospective safety trials. Because of these factors, DPP-4 inhibitors have become an established therapy for T2DM and are increasingly being positioned earlier in treatment algorithms [39]. When sulfonylureas are used with respect to very low cost, Gliclazide should be preferred versus other sulfonylureas based on the lower risk for hypoglycaemia and better CV safety profile [47–50]. The glucose-lowering potency is very similar for most of the anti-diabetic drugs when starting at a HbA1c level of about 8 % [51], however when HbA1c values are higher Insulin, GLP-1 receptor agonists, or SGLT2-inhibitors are more powerful than DPP-4 inhibitors and sulfonylureas [52–54].

In T2DM patients already presenting with CVD, principally all drugs (DPP-4 inhibitors, GLP-1 receptor agonists, and basal insulin glargine) with confirmed safety in outcome studies [16–20] could be used, however in order to reduce CV events and CV death, a combination of drugs should be preferred with documented CV benefit. The triple combination of metformin, pioglitazone, and empagliflozin seems to be at the moment the best option (Table 2) to reduce the high risk for recurrent myocardial infarction, acute coronary syndrome or stroke in patients with a history of CVD [22, 23]. In addition, such a combination would result in reduction of CV death and all-cause mortality by about one third [21]. This triple combination would be very effective in lowering HbA1c by different mechanisms – reduction of hepatic glucose production, improvement of insulin sensitivity and by the glucoretic effect [54–58] – but not inducing any risk of hypoglycaemia and offering weight neutrality. The profound effect of lowering of both BP and albuminuria – mediated by different mechanism – may be helpful to reduce the vascular burden of the high risk patients [41, 42, 58, 59]. This triple combination could also be used in patients with CKD stages 3 and 4, since a significant reduction of CV events/mortality was documented for all three compounds, for metformin [60, 61], pioglitazone [25], and empagliflozin [35]. In the presence of heart failure pioglitazone has to be stopped [31], although the well-known water retention effect of pioglitazone may be neutralized by empagliflozin (Table 2). Table 2 shows that (a) some positive or negative effects of the three individual  drugs may be neutralized in combination; and (b) in addition some positive effects could also work synergistically. Unfortunately, no study will ever be done to prove our treatment concept for diabetic patients already presenting with CVD. Since in some but not in all studies DPP-4 inhibitors were associated with an increased the risk for heart failure [62–66], these compounds may also not be used in patients with concomitant occurrence of CVD and heart failure. A recent study showed that the risk for heart failure is particularly high in the presence of CKD [67], thus patients with CVD, CKD and heart failure should be treated with SGLT2 inhibitors but not with DPP-4 inhibitors [66].

**Table 2:** Anticipated combinatory effect of metformin, pioglitazone, and empagliflozin

	Metformin	Pioglitazone	Empagliflozin	Anticipated effect?
Cardiovascular death	↓	↔	↓↓	↓↓↓
All-cause death	↓	↔	↓↓	↓↓↓
Myocardial infarction	↓	↓	↔	↓↓
Stroke	↓	↓	↔	↓↓
Peripheral arterial disease	↓	↔	↔	↓
Fluid retention	↔	↑	↓	↔
Heart failure	↔	↑	↓	↔
Weight	↓	↑	↓	↓
Blood pressure	↔	↓	↓	↓↓
HbA1c	↓	↓	↓	↓↓↓
LDL-cholesterol	↓	↔	↑	↔
HDL-cholesterol	↔	↑	↑	↑↑
Albuminuria	↔	↓	↓	↓↓
Insulin sensitivity	↑	↑↑	↑	↑↑↑↑

↓ lowered, ↑ elevated, ↔ unchanged

In summary, prevention of CV morbidity and mortality remains to be the key factor in the treatment of T2DM. In the early phase of T2DM multifactorial intervention is mandatory and glucose levels should be near normal, in particular in the younger patients presenting with the highest long-term CV risk. Anti-diabetic drugs without any risk for hypoglycaemia should be preferred in order to reduce clinical inertia and increase long-term adherence to the treatment. Two very recent studies [68, 69] are not in favour of a wide use of sulfonylureas or insulin. In a nationwide study [68] using Taiwan’s National Health Insurance Research Database, DPP-4 inhibitors were associated with lower risks for all-cause death (HR 0.63 [95 % CI 0.55–0.72]), MACE (HR, 0.68 [95 %CI 0.55–0.83]), ischemic stroke (HR, 0.64 [95 %CI 0.51–0.81]), and hypoglycaemia (HR, 0.43 [95 %CI 0.33–0.56]) compared with sulfonylureas as add-on therapy to metformin but had no effect on risks for myocardial infarction and hospitalization for heart failure. A recent meta-analysis [69] of randomized controlled trials evaluating the effects of insulin versus oral hypoglycaemic agents (OHAs) on all-cause mortality and CV outcomes in patients with T2DM did not show any superiority for insulin therapy concerning all-cause mortality (RR = 1.00; 95 % CI 0.93–1.07), CV death (RR = 1.00; 95 % CI 0.91–1.09), myocardial infarction (RR = 1.04; 95 % CI 0.93–1.16), angina (RR = 0.97; 95 % CI 0.88–1.06), sudden death (RR = 1.02; 95 % CI 0.66–1.56), or stroke (RR = 1.01; 95 % CI 0.88–1.15). However, insulin reduced the risk of heart failure compared with OHAs (RR = 0.87; 95 % CI 0.75–0.99). In the very high risk subgroup of secondary prevention of CVD insulin did not differ from OHAs in all-cause mortality, CV death, myocardial infarction, or stroke.

In patients presenting with CVD the best outcome may be expected with the triple oral therapy of metformin, pioglitazone and empagliflozin, although a controlled prospective study versus insulin therapy is needed to confirm the expectation.

Our recommendation for the inclusion of pioglitazone in the triple therapy in diabetic patients with a history of CVD is strongly supported by recent findings of the IRIS study [70, 71], where pioglitazone or placebo were added to nondiabetic patients after ischaemic stroke or TIA. After a treatment period of 4.8 years pioglitazone prevented stroke or myocardial infarction by 24 % relative risk reduction and by 2.9 % absolute risk reduction (*p* < 0.007), despite the fact that the patients were well treated according to current guidelines (antiplatelet drugs in 95 %, statins in 82 %); blood pressure values were 133/79 mmHg and LDL values 89 mg/dl. In the insulin-resistant nondiabetic patients diabetes developed in 7,7 % of the patients receiving placebo, but in only 3.8 % under pioglitazone (58 % relative risk reduction, *p* < 0.001). Incidence of heart failure or incident cancer were not increased in patients receiving pioglitazone, but there was more weight gain (plus 3 kg) and bone fractures under pioglitazone compared with placebo (5.1 vs. 3.2 %, *p* = 0.01). Since two thirds of nondiabetic patients with CVD and almost all patients with overt diabetes are insulin resistant, these data show for the first time that a therapy directed to insulin resistance can prevent cardiac and cerebrovascular events and has important clinical consequences.
